# Comparison of the efficacy and safety of ciprofol and propofol in sedating patients in the operating room and outside the operating room: a meta-analysis and systematic review

**DOI:** 10.1186/s12871-024-02609-3

**Published:** 2024-07-02

**Authors:** Yanni Yang, Zekun Lang, Xiumei Wang, Peining Yang, Ning Meng, Yang Xing, Yatao Liu

**Affiliations:** 1 Department of Anesthesiology and Operating Theater, The First Hospital of Lanzhou University, No.1 Donggang west Road, Chengguan District, Lanzhou, Gansu 730000 China; 2 The First Clinical Medical College of Lanzhou University, Lanzhou, Gansu 730000 China

**Keywords:** Ciprofol, Propofol, Anesthesia, Sedation

## Abstract

**Background:**

As a new type of intravenous anesthetic, ciprofol has the advantages of fast onset of action, fast recovery and high clearance rate. This study aimed to investigate the effectiveness and safety of ciprofol versus traditional propofol for anesthesia and sedation in and out of the operating room.

**Methods:**

We searched the literature in PubMed, Web of Science, Cochrane Library, and Embase databases from January 2021 to December 2023. All clinical studies comparing the sedative effects of propofol and ciprofol, both inside and outside the operating room, were included in our trial. The main outcome measures were induction time and incidence of injection-site pain. Data are merged using risk ratio and standardized mean difference with 95% confidence interval. Subgroup analysis, meta-regression, sensitivity analysis, and publication bias were performed. The study protocol was prospectively registered with PROSPERO (CRD42023447747).

**Results:**

A total of 15 randomized, controlled trials involving 2002 patients were included in this study. Compared with propofol, ciprofol has a longer induction time in the operating room but a shorter induction time in non-operating room settings. Ciprofol can effectively reduce the risk of injection-site pain and respiratory depression both inside and outside the operating room. In addition, the risk of drug-related hypotension induced with ciprofol in the operating room is lower, but the awakening time is also longer. Meta-regression analysis showed that neither age nor BMI were potential sources of heterogeneity. Funnel plot, egger and begg tests showed no significant publication bias. Sensitivity analyzes indicate that our results are robust and reliable.

**Conclusion:**

Ciprofol has absolute advantages in reducing the risk of injection-site pain and respiratory depression, both in and outside operating room. Intraoperative use of ciprofol reduces the risk of drug-related hypotension and may also reduce the risk of intraoperative physical movements. However, ciprofol may have longer induction and awakening time than propofol.

**Supplementary Information:**

The online version contains supplementary material available at 10.1186/s12871-024-02609-3.

## Introduction

Propofol is a drug widely used in the field of anesthesia and sedation. It has been used for a long time until now to induce and maintain anesthesia during surgery, as well as sedation during medical operations and examinations [1–3]. As a short-acting intravenous anesthesia drug, propofol acts quickly, and the patient wakes up quickly from anesthesia, but propofol may also cause adverse reactions such as injection-site pain, hypotension, respiratory depression, and slow heart rate [4–6]. Ciprofol is a novel short-acting γ-aminobutyric acid (GABA) receptor agonist with anesthetic and sedative effects [7]. Similar to propofol, it has the characteristics of quick onset and quick recovery, but ciprofol has less pain after injection and stable cardiopulmonary function. Since ciprofol is a newly developed drug in recent years, although there are many studies underway, the published studies are very limited. Therefore, we designed and implemented this meta-analysis and systematic review as a high-quality evidence-based medical evidence to guide subsequent research and clinical work.

## Methods

The report of this meta-analysis follows the guidelines of the PRISMA statement (Preferred Reporting Items for Systematic Reviews and Meta-Analyses). The PRISMA Statement is an initiative to improve the quality of reporting of systematic reviews and meta-analyses, helping to ensure transparency and credibility of research through clear reporting norms and checklists [8]. This study has been registered in the international registration database PROSPERO (CRD42023447747). PROSPERO is an international database dedicated to registration system reviews and Meta-analysis, which aims to improve the transparency and traceability of research.

### Data sources and search strategy

We searched PubMed, Web of Science, Cochrane Library, and Embase from January, 2021 to December, 2023, and supplemented the literature of China national knowledge infrastructure. All database searches are based on the combination of subject terms and free words to construct search terms, and make appropriate adjustments according to specific databases. The search query consists roughly of the following: propofol in mesh AND ciprofol in Title or Abstract. The specific search strategy is presented in the supplementary material in word form.

### Study selection

In the first screening, the two authors (WXM and YPN) independently selected the literature related to this study by reading the titles and abstracts of the literatures, and then retrieved and read the full text to determine the studies that finally met the inclusion criteria. Disagreement is resolved through discussion with a third party (YYN and LYT). All included studies were randomized controlled trials comparing the effectiveness and safety of propofol and ciprofol in different settings, and each study reported the outcomes to be studied in this meta-analysis.

The exclusion criteria are as follows: (i) patients with severe circulatory system diseases (uncontrolled hypertension, severe arrhythmia, chronic heart failure, unstable angina, myocardial infarction within 6 months, etc.), respiratory system diseases (respiratory failure, chronic obstructive pulmonary disease, etc.) are excluded. Patients with acute exacerbation, bronchospasm or acute respiratory infection requiring treatment within the past 3 months and with a history of obvious fever, wheezing, nasal congestion and cough), or patients with a history of drug abuse or allergies. Studies of patients with dyspneic or apneic syndromes will also be excluded. (ii) Studies lacking relevant data for inclusion in the analysis will also be excluded. (iii) Animal studies, conference abstracts, correspondence, protocols, case reports, duplications and other non-randomized studies will be excluded.

### Data collection and quality evaluation

One reviewer (YYN) extracted data for each included study, and another reviewer (LZK) ensured its accuracy and completeness. Any discrepancies were resolved through discussion, and if needed, a third reviewer (LYT) was consulted. First, we extracted relevant baseline data, such as sample size, age, body mass index (BMI), duration and type of surgery, and other drugs used for induction. In addition, we also extracted data related to outcomes, such as induction time, injection-site pain and hypotension and bradycardia related to drug during operation, etc.

In accordance with the “Randomized Trial Bias Risk Assessment Tool” in the Cochrane Handbook, an assessment is conducted to evaluate the quality of the literature that has been included [9]. The assessment encompasses seven areas: allocation hiding, randomization method, blinding method between investigator and subject, blinding method of result evaluator, and selective reporting of results, data completeness, and other possible biases.

We also employed the Grading of Recommendations, Assessment, Development, and Evaluation (GRADE) system to evaluate the quality of evidence. Utilizing the GRADE methodology, each outcome was categorized as ‘high,’ ‘moderate,’ ‘low,’ or ‘very low’ in terms of evidence quality. According to GRADE, randomized controlled trials (RCTs) start with the highest level of evidence. Nevertheless, this level of evidence may be reduced based on limitations observed in five key aspects: risk of bias, consistency, indirectness, imprecision, and publication bias [10].

### Outcomes and definitions

The main outcomes are induction time and injection-site pain. The definition of induction time is basically from the beginning of administration to the BIS value falling below 60, or according to the Modified Observer’s Assessment of Alertness/Sedation (MOAA/S) scale [11–13], from the beginning of administration to MOAA/S ≤ 1. Secondary outcomes included hypotension and bradycardia related to drug during operation, awakening time, respiratory depression, time to disappearance of eyelash reflex or loss of consciousness and body moving during operations.

### Statistical analysis

Data analysis was conducted using Review Manager 5.4 and Stata SE 16.0 (Stata Corporation, Texas, USA). For dichotomous data, we estimated the risk ratio (RR) with 95% confidence intervals (CI), and for continuous data, the standard mean difference (SMD) with 95% CI was calculated. To address both methodological and clinical heterogeneity, we utilized both fixed and random effect models for data synthesis [9]. Heterogeneity was evaluated using the Q-test and I^2^ statistic, considering significance at *p* < 0.1 or I^2^ > 50%. Meta-regression analyses were performed to investigate potential sources of heterogeneity. Evaluation of publication bias risk was conducted using funnel plots, and egger and begg test was applied when a minimum of 10 studies were available. All analyses were carried out with a significance level of α = 0.05. Sensitivity analysis was conducted to assess result robustness and identify sources of heterogeneity.

## Results

### Study selection

**Fig. 1:**
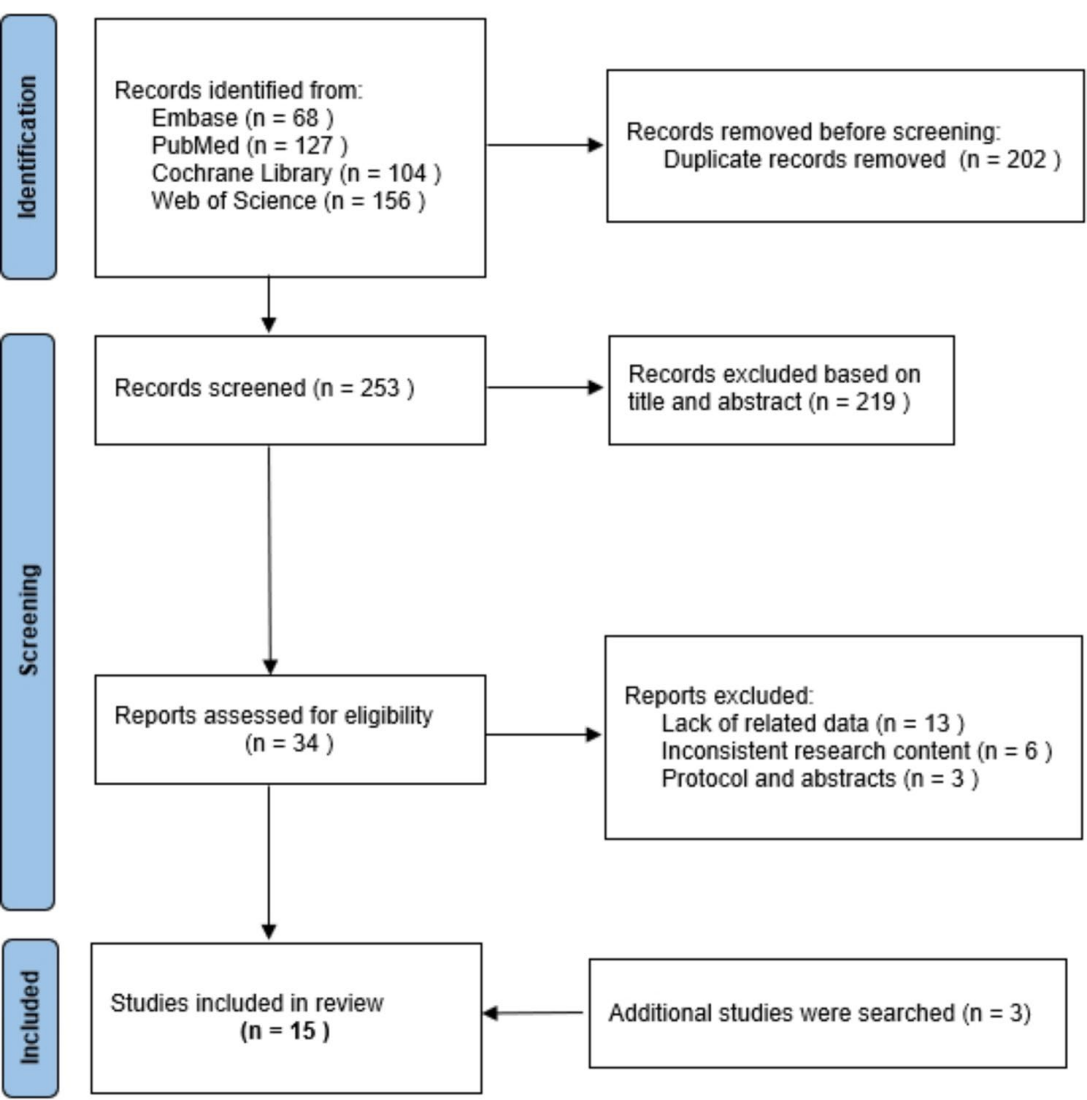
Flowchart of selection according to the Preferred Reporting Items for Systematic Reviews and Meta-Analyses (PRISMA) guidelines

From PubMed, Web of Science, Cochrane Library, and Embase, we initially identified 455 pertinent studies. After removing duplicate documents, 253 studies remained. Upon reviewing the abstracts and titles, we identified 219 studies that did not align with our research objectives. Following a thorough examination of the full text of the remaining 34 documents, we excluded 22 studies. Among these, 3 studies exhibited inconsistencies in content and form, 13 lacked relevant data, and 6 diverged from our research direction. Additionally, through a supplementary search of 3 high-quality Chinese studies, we ultimately included a total of 15 trials [14–28]. Figure 1 illustrates the detailed screening procedure.

### Baseline characteristics and study assessment

**Fig. 2:**
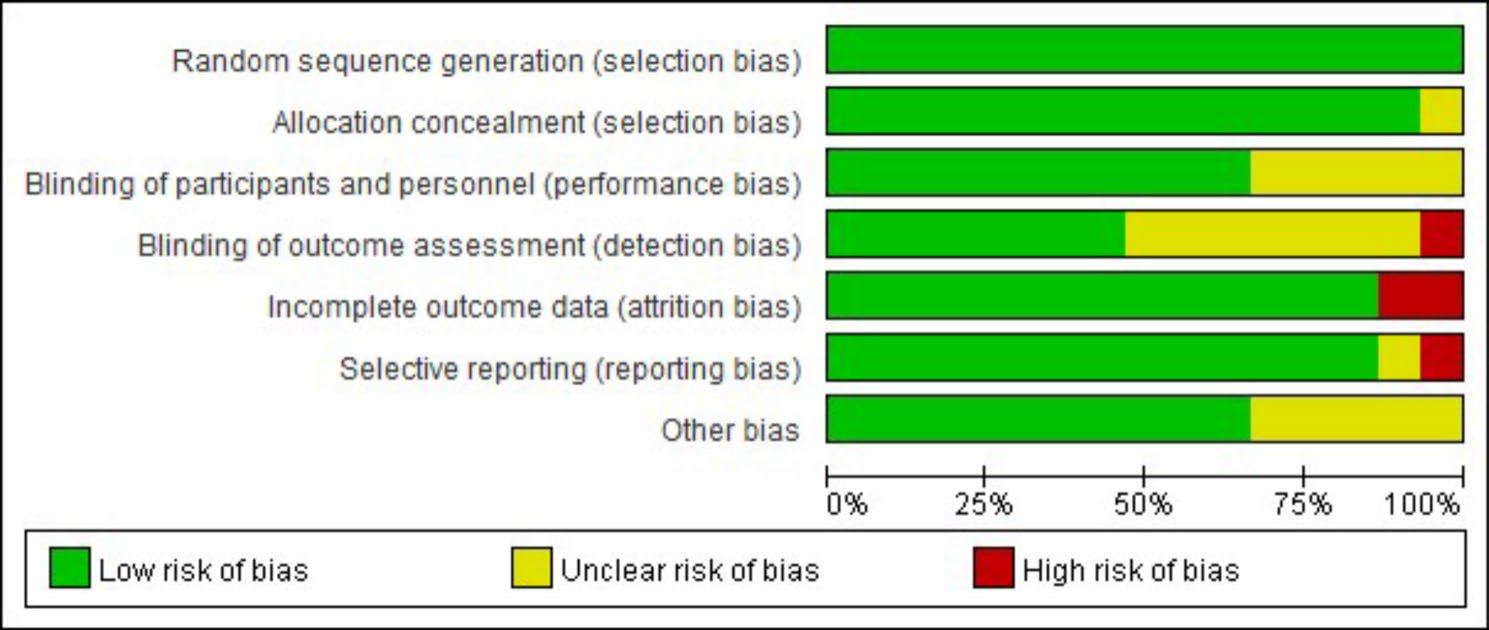
Risk of bias graph: review authors’ judgements about each risk of bias item presented as percentages across all included studies

**Fig. 3:**
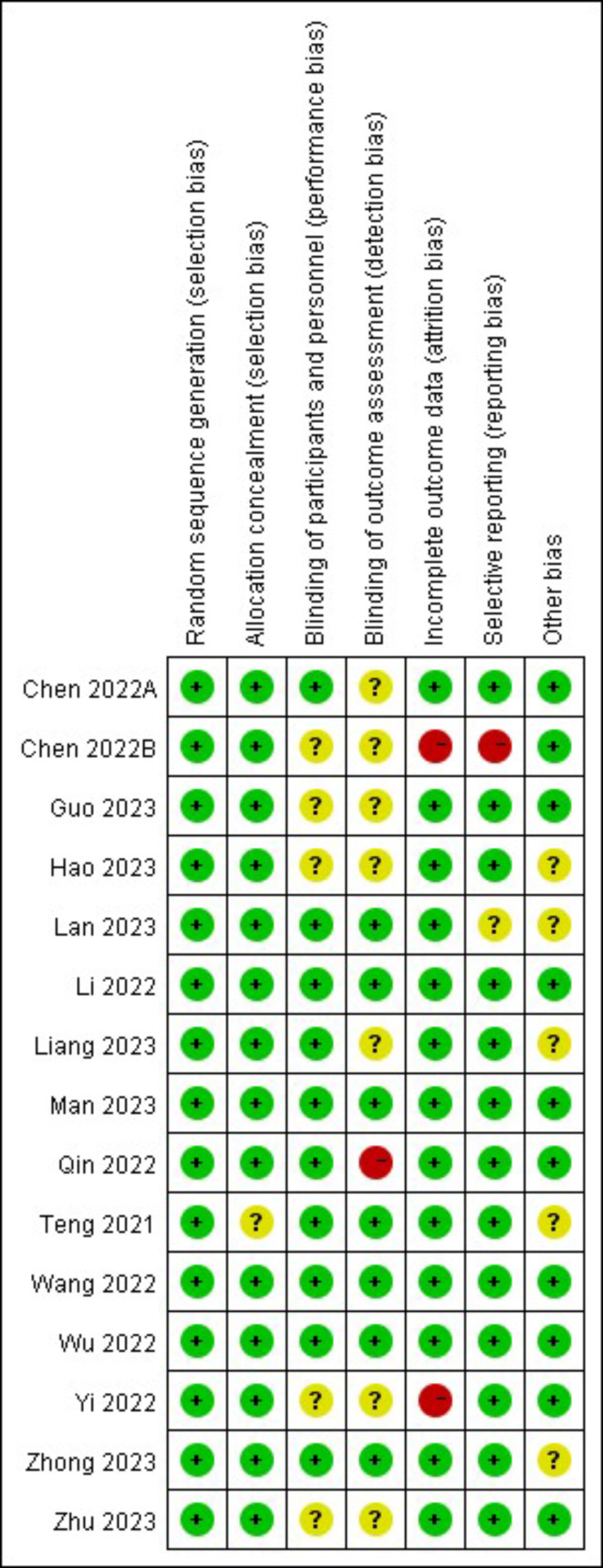
Risk of bias summary: review authors’ judgements about each risk of bias item for each included study

These studies are all conducted after 2021, with sample sizes ranging from 64 to 289. A total of 2002 patients participated in this study, with an average age of 47.1 ± 10.2. Basic information about the included studies is summarized in Tables 1 and 2. Figures 2 and 3 provide an overview of the risk of bias assessment for each included trial. Overall, 4 studies were evaluated as low risk [19, 21, 24, 25], 8 studies were evaluated as having uncertain risk [14, 16–18, 20, 23, 27, 28], and 3 studies were evaluated as high risk [15, 22, 26]. The GRADE evaluation results are shown in Table 3.

**Table 1:** Basic information about studies in the operating room

Study	Sample size	Age (year)	BMI (kg/m^2^)	Operative duration (min)	Other drugs used for induction	Type of surgery
Chen [14]	120	33.9 ± 9.4	21.8 ± 3.0	53.3 ± 21.8	MZ (0.03 mg/kg); SF (0.3 µg/kg); RC (0.6 mg/kg)	Gynecological surgery
Guo [16]	80	50.1 ± 9.6	23.9 ± 2.9	75.5 ± 10.7	SF (0.5 µg/kg); RC (0.6 mg/kg)	Laparoscopic cholecystectomy
Hao [17]	100	51.1 ± 10.2	24.0 ± 2.4	37.5 ± 9.1	AF (5 µg/kg)	Hysteroscopic surgery
Lan [18]	149	42.7 ± 11.3	23.3 ± 2.7	21.1 ± 9.5	SF (0.4 µg/kg)	Hysteroscopic surgery
Liang [20]	128	39.2 ± 10.1	23.3 ± 2.9	94.3 ± 38.0	MZ (0.04 mg/kg); SF (0.3 µg/kg); RC (0.6 mg/kg)	Elective surgery
Man [21]	128	43.2 ± 9.4	23.1 ± 2.4	NA	MC (0.2 mg/kg); AF (20ug/kg)	Gynecological surgery
Qin [22]	105	40.1 ± 10.4	23.2 ± 2.9	170.6 ± 38.2	SF (0.4–0.5 µg/kg); CA (0.2 mg/kg)	Kidney transplantation
Wang [24]	176	39.8 ± 11.6	23.3 ± 3.0	NA	MZ (0.04 mg/kg); SF (0.3 µg/kg); RC (0.6 mg/kg)	Elective surgery
Zhu [28]	109	46.1 ± 12.4	24.2 ± 2.8	83.1 ± 49.4	MZ; SF; RC	Elective surgery

BMI, Body Mass Index; MZ, midazolam; SF, sufentanil; RC, rocuronium; AF, alfentanil; MC, mivacurium chloride; CA, cisatracurium; NA, not acceptable

**Table 2:** Basic information of studies on non-operating room settings

Study	Sample size	Age (year)	BMI (kg/m^2^)	Female	Other drugs related to anesthesia	Purpose
Chen [15]	96	42.2 ± 12.0	24.3 ± 6.7	57	None	Gastroenteroscopy
Li [19]	289	44.0 ± 11.6	23.3 ± 2.6	171	Fentanyl (50 µg)	Gastroenteroscopy
Teng [23]	64	45.1 ± 9.8	23.1 ± 2.5	NA	Fentanyl (50 µg)	Gastroenteroscopy
Wu [25]	92	57.8 ± 5.4	24.4 ± 1.9	42	Fentanyl (50 µg)	FB
Yi [26]	159	69.9 ± 2.9	23.7 ± 3.1	76	SF (0.1 µg/kg)	Gastroenteroscopy
Zhong [27]	207	57.3 ± 13.1	22.5 ± 2.9	96	RF and ET	ESD, ERCP, and FB

BMI, Body Mass Index; NA, Not Acceptable; FB, flexible bronchoscopy; SF, sufentanil; RF, remifentanil; ET, esketamine; ESD, endoscopic submucosal dissection; ERCP, endoscopic retrograde cholangiopancreatography

**Table 3:** GRADE evidence summary table

No. of studies	Trial design	Quality assessment					No. of patients		Effect		Quality of the evidence
		RoB	Inconsistency	Indirectness	Imprecision	PB	Ciprofol	Propofol	Relative (95%CI)	Absolute (95%CI)	
**Induction time**											
10	RCT	Serious^a^	No	No	No	No	707	776	-	SMD **0.32 higher** (0.42 lower to 1.06 higher)	⊕ ⊕ ⊕ ⊝Moderate
**Incidence of injection-site pain**											
15	RCT	No	No	No	No	No	60/1102 (5.4%)	406/931 (43.6%)	**RR 0.13** (0.10 to 0.17)	**379 fewer per 1000** (from 362 fewer to 392 fewer)	⊕ ⊕ ⊕ ⊕High
**Hypotension related to drug**											
12	RCT	No	No	No	No	No	273/871 (31.3%)	359/871 (41.2%)	**RR 0.78** (0.61 to 1.00)	**91 fewer per 1000** (from 161 fewer to 0 more)	⊕ ⊕ ⊕ ⊕High
**Bradycardia related to drug**											
12	RCT	Serious^b^	No	No	No	No	142/936 (15.2%)	99/798 (12.4)	**RR 1.02** (0.81 to 1.28)	**2 more per 1000** (from 24 fewer to 35 more)	⊕ ⊕ ⊕ ⊝Moderate
**Awakening time**											
10	RCT	No	No	No	No	No	664	651	-	SMD **0.1 higher** (0.22 lower to 0.42 higher)	⊕ ⊕ ⊕ ⊕High
**Respiratory depression**											
10	RCT	No	No	No	No	No	69/814 (8.5%)	115/675 (17.0%)	**RR 0.51** (0.38 to 0.67)	**83 fewer per 1000** (from 56 fewer to 106 fewer)	⊕ ⊕ ⊕ ⊕High
**Time to disappearance of eyelash reflex**											
6	RCT	Serious^c^	No	No	No	No	429	360	-	SMD **0.5 higher** (0.33 lower to 1.32 higher)	⊕ ⊕ ⊕ ⊝Moderate
**Body moving**											
5	RCT	No	No	No	No	No	45/328 (13.7%)	61/328 (18.6%)	**RR 0.74** (0.54 to 1.01)	**48 fewer per 1000** (from 86 fewer to 2 more)	⊕ ⊕ ⊕ ⊕High

RoB, Risk of bias; PB, Publication bias; SMD, Standardized Mean Difference ; RR, risk ratio

^a^ The included studies may have attrition bias

^b^ The included studies may have detection bias

^c^ The included studies may have reporting bias

### Main outcomes of the meta-analysis

**Fig. 4:**
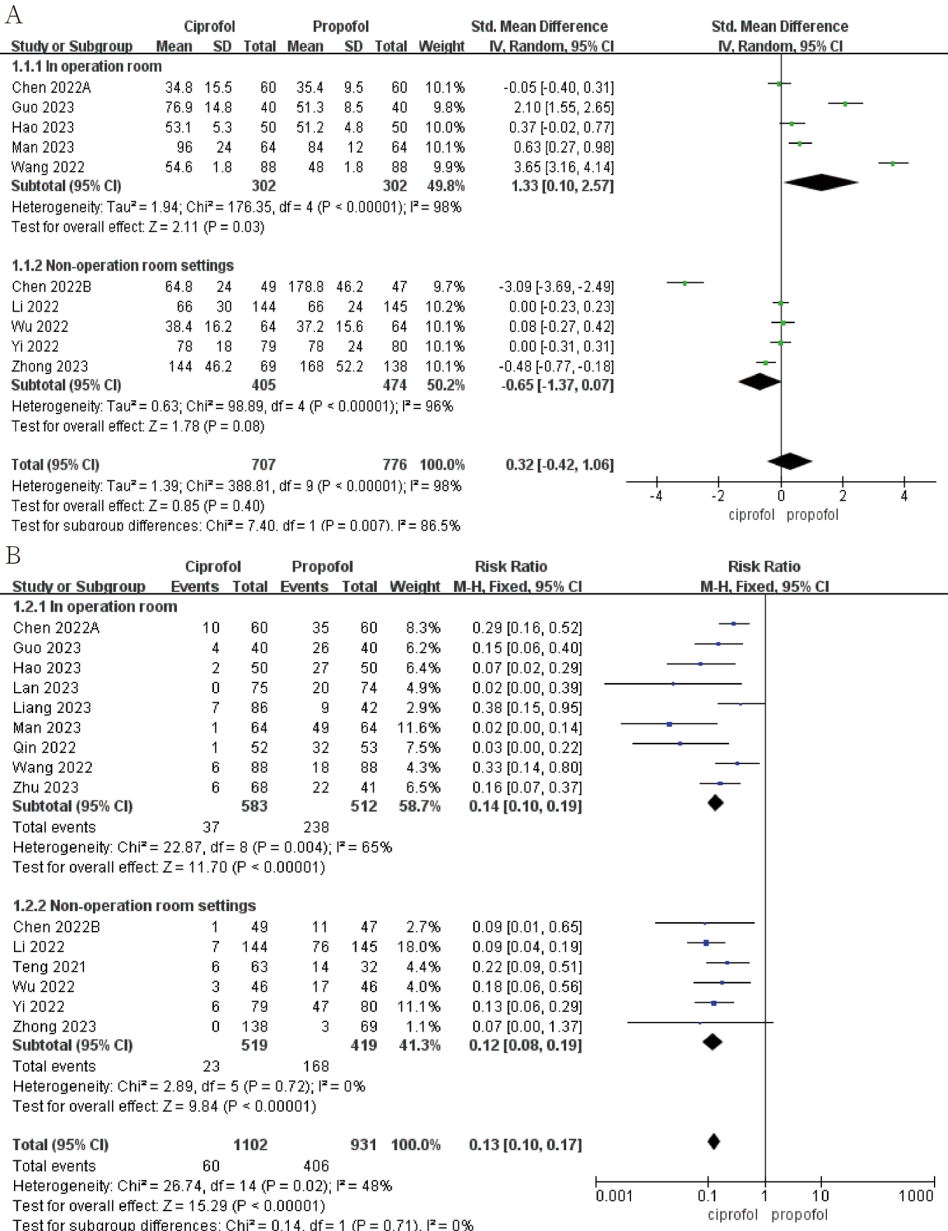
The pooled results of (**A**) Induction time. (**B**) Incidence of injection-site pain of propofol and ciprofol in and outside the operating room

The results show that propofol induction time is shorter for conventional surgery (Fig. 4A; SMD, 1.33; 95%CI, 0.10 to 2.57; *p* = 0.03; I^2^ = 98%), while ciprofol induction time is shorter in non-operating room settings (Fig. 4A; SMD, -0.65; 95%CI, -1.37 to 0.07; *p* = 0.08; I^2^ = 96%). Additionally, almost all studies show that ciprofol has a lower incidence of injection-site pain (Fig. 4B; RR, 0.13; 95%CI, 0.10 to 0.17; *p*<0.00001; I^2^ = 48%), both for surgery (Fig. 4B; RR, 0.14; 95%CI, 0.10 to 0.19; *p*<0.00001; I^2^ = 65%) and in non-operating room settings (Fig. 4B; RR, 0.12; 95%CI, 0.08 to 0.19; *p*<0.00001; I^2^ = 0).

**Fig. 5:**
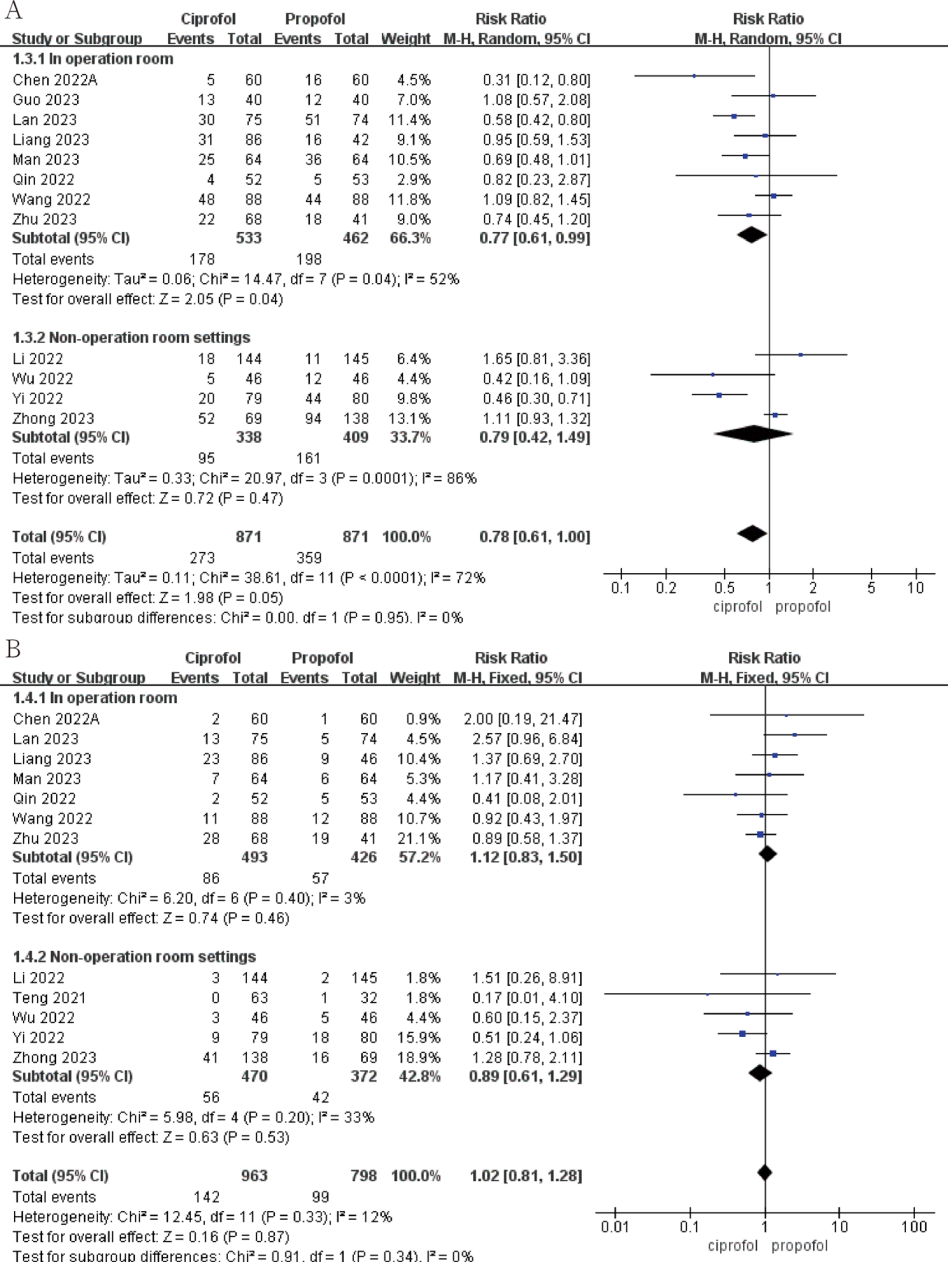
The pooled effect of (**A**) Hypotension related to drug during operation. (**B**) Bradycardia related to drug during operation of propofol and ciprofol in and outside the operating room

**Fig. 6:**
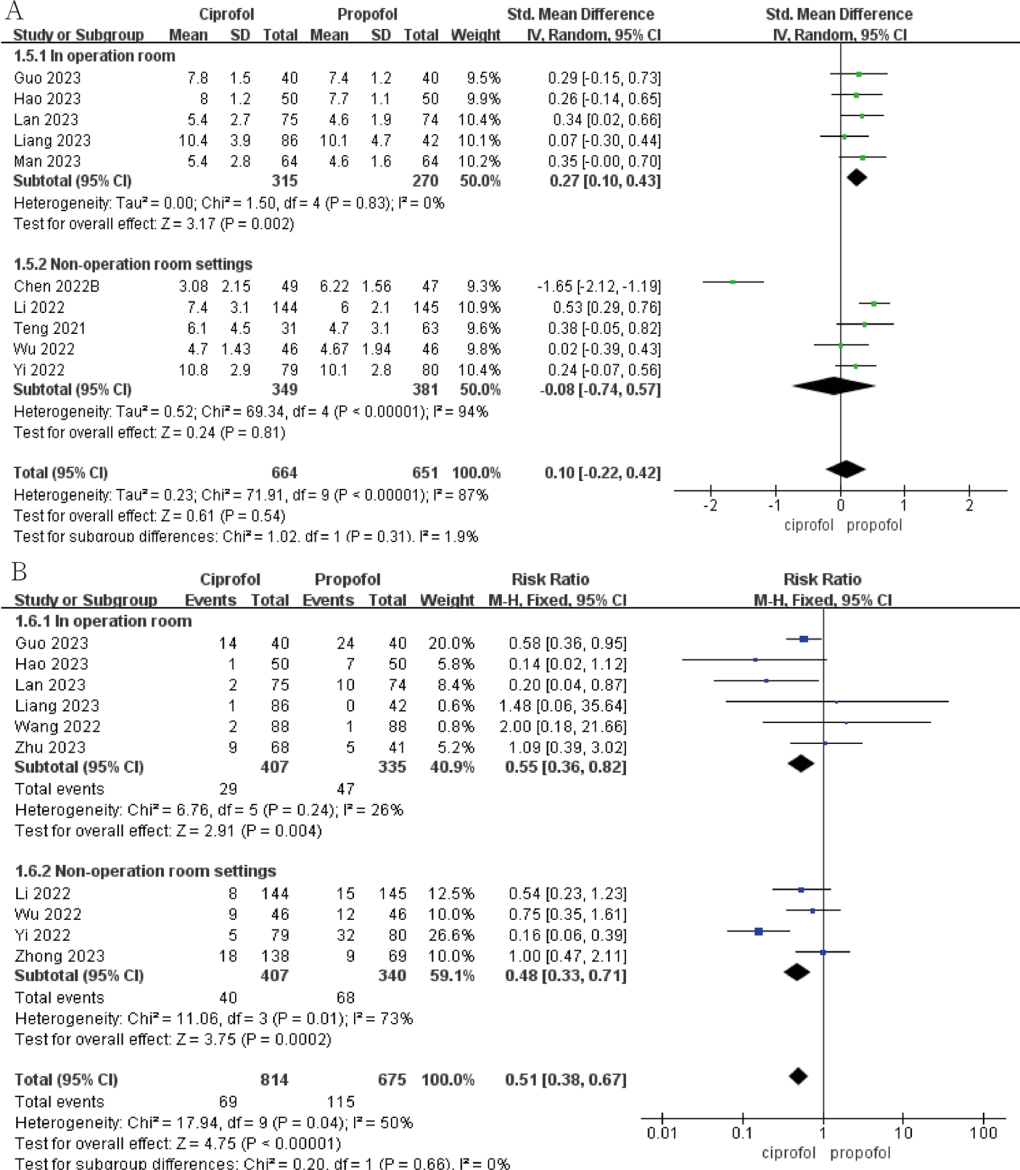
The pooled effect of (**A**) Awakening time. (**B**) Respiratory depression of propofol and ciprofol in and outside the operating room

**Fig. 7:**
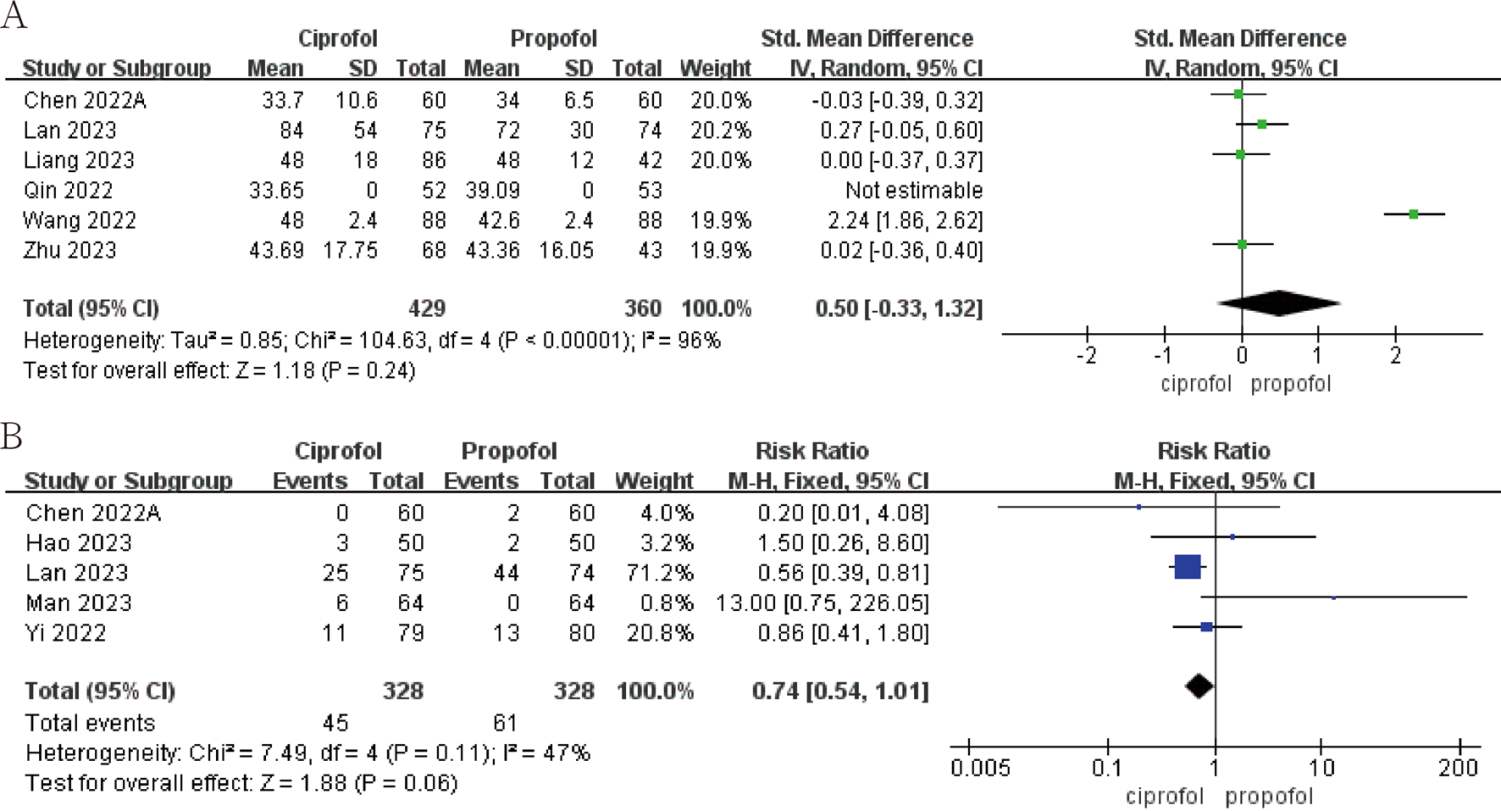
The pooled effect of (**A**) Time to disappearance of eyelash reflex or loss of consciousness. (**B**) Body moving during operation of propofol and ciprofol in the operating room

Ciprofol has a lower incidence of intraoperative drug-related hypotension than propofol (Fig. 5A; RR, 0.77; 95%CI, 0.61 to 0.99; *p* = 0.04; I^2^ = 52%). Ciprofol also had a lower incidence of drug-related hypotension than propofol in the non-operating room setting, although this was not statistically significant (Fig. 5A; RR, 0.79; 95%CI, 0.42 to 1.49; *p* = 0.47; I^2^ = 86%). The incidence of drug-related bradycardia with ciprofol is higher than propofol during surgery, but the incidence of drug-related bradycardia with ciprofol is lower than propofol in the non-surgical settings, this result is not statistically significant (Fig. 5B). Statistically significant results show that propofol has a shorter awakening time than ciprofol during surgery (Fig. 6A; SMD, 0.27; 95%CI, 0.10 to 0.43; *p* = 0.002; I^2^ = 0). In addition, ciprofol has a lower incidence of respiratory depression both during surgery (Fig. 6B; RR, 0.55; 95%CI, 0.36 to 0.82; *p* = 0.004; I^2^ = 26%) and in non-operating room settings (Fig. 6B; RR, 0.48; 95%CI, 0.33 to 0.71; *p* = 0.0002; I^2^ = 73%). Finally, ciprofol has a longer time to disappearance of eyelash reflex and a lower incidence of body moving during operation in operation room, but neither result was statistically significant (see Fig. 7).

### Subgroup analysis

**Fig. 8:**
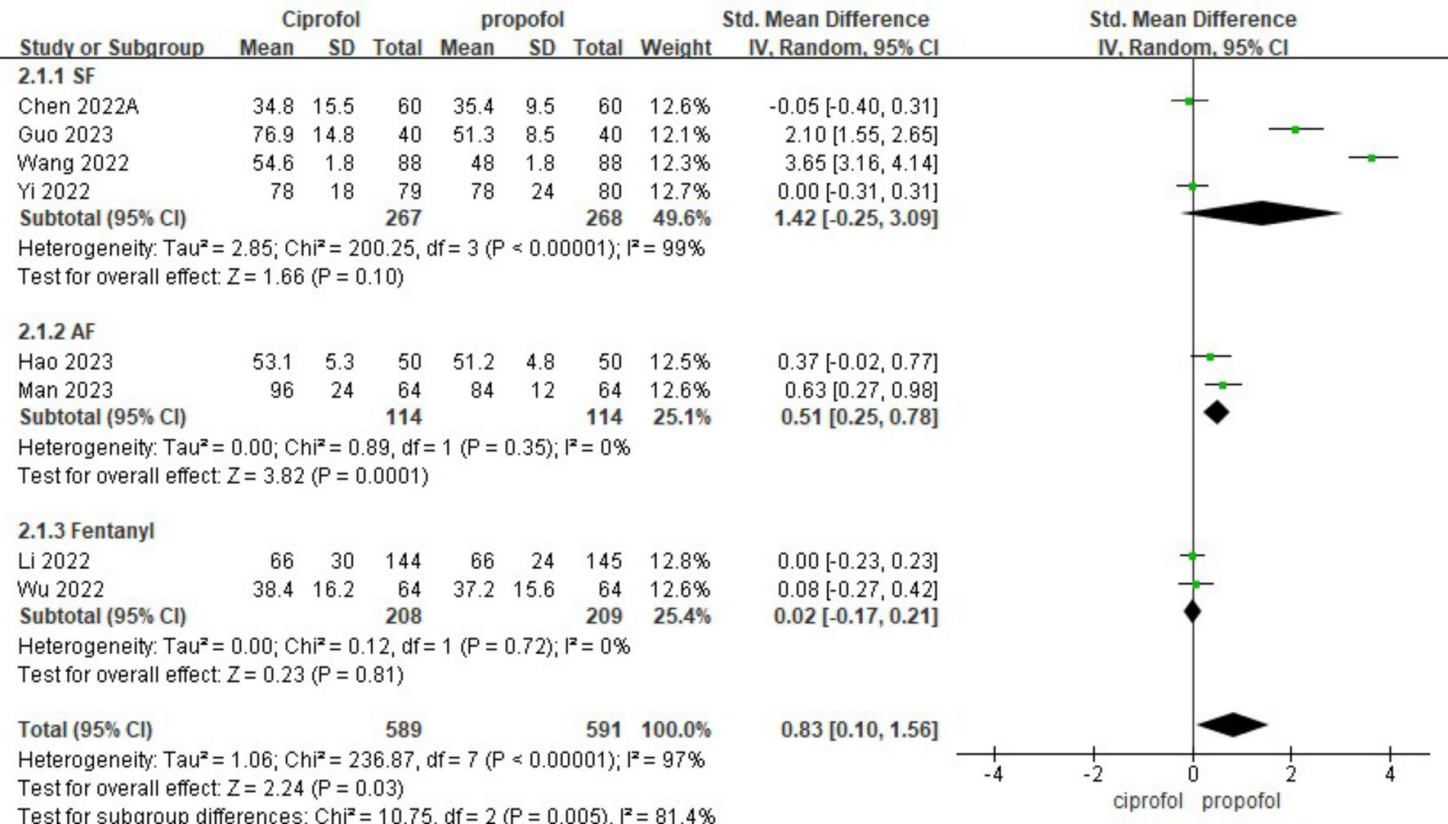
Subgroup analysis of different analgesics for induction time

**Fig. 9:**
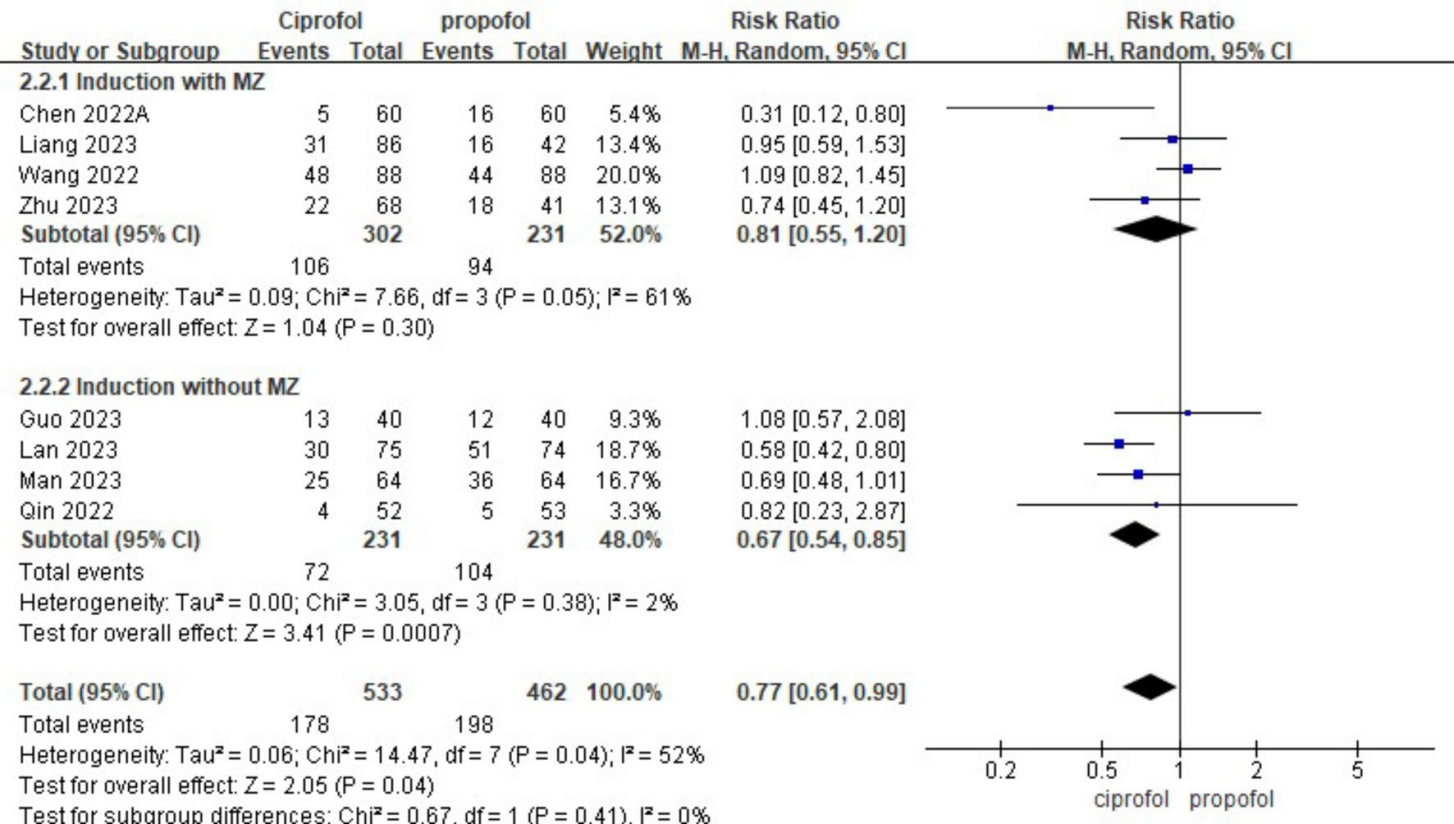
Subgroup analysis of midazolam use regarding hypotension related to drug during operation

We conducted a subgroup analysis regarding the utilization of various analgesic medications during induction. Our findings revealed significant heterogeneity among the groups, suggesting that the use of different analgesic drugs during induction could be a potential source of this variability (Fig. 8). We performed a subgroup analysis to assess the impact of midazolam administration during drug-induced hypotension in surgical procedures. Our findings revealed no significant heterogeneity across the groups. Irrespective of midazolam usage during induction, individuals in the ciprofol group exhibited a lower risk of developing hypotension (Fig. 9).

### Meta-regression

Age and BMI were included in random-effect univariate meta-regression analyzes for induction time, incidence of injection-site pain, incidence of hypotension related to drug during operation and respiratory depression. The results indicate that Age and BMI are not the main sources of heterogeneity in these outcomes. Specific details are presented in the supplementary material.

### Publication bias assessment and sensitivity analysis

The funnel plots for induction time, incidence of injection-site pain, hypotension related to drug during operation, bradycardia related to drug during operation, awakening time, and respiratory depression show no notable publication bias. All funnel plots are provided in the supplementary materials. We also conducted Egger’s and Begg’s tests, which indicated that our findings were not influenced by significant publication bias. Detailed results can be found in the supplementary material. For other outcomes, given the limited number of included studies, neither Begg’s nor Egger’s test was conducted. However, sensitivity analysis reveals that the aggregated results remain robust and dependable. Detailed results of the sensitivity analysis are provided in the supplementary material.

## Discussion

Ciprofol is a propofol analog independently developed in China and is formulated with an injectable emulsion composed of medium and long-chain triglycerides. Ciprofol, like propofol, exerts its sedative and hypnotic effects through GABA receptors. But ciprofol has higher fat solubility and potency than propofol [29]. Ciprofol is metabolized by glucose catalysis, oxidation and sulfation into harmless glucuronic acid conjugates and is primarily excreted from the kidneys. Ciprofol begins to take effect 1 to 2 min after administration and gradually recovers within 10 to 18 min, indicating that Ciprofol takes effect quickly. In addition, in clinical applications, we found that the subjects recovered smoothly and quickly, and the elimination half-life was 1.58 to 2.47 h. Currently, ciprofol is approved for the induction and maintenance of anesthesia in adults, as well as for sedation and anesthesia during non-tracheal intubation procedures [7, 30, 31].

Our research shows that ciprofol has an absolute advantage in the incidence of injection-site pain, which is also consistent with our experience. Surprisingly, propofol had a shorter induction time. Different studies have reported different results, with Guo et al [16]. and Wang et al [24]. both reporting that propofol has a shorter induction time. Lan et al [18]. and Liang et al [20]. reported that ciprofol has a shorter induction time. We believe that both propofol and ciprofol have a relatively rapid onset of action, so there is no significant difference in the induction time between the two. In addition, this difference may be caused more by the speed at which anesthesiologists push drugs and individual differences among patients. While certain results exhibited considerable heterogeneity, meta-regression analysis indicated that patient age and BMI did not contribute significantly to this variability. We posit that clinical heterogeneity, particularly variances in drug dosage and adjunctive medication types, constitutes the primary source of heterogeneity. Additionally, divergent surgical procedures may also contribute to this variability. In conclusion, we contend that our findings hold meaningful reference value.

Propofol stands out as the prevailing sedative-hypnotic drug in current medical practice, primarily employed for anesthesia induction and sedation of patients in intensive care units. Since the late 1980s, propofol has gained extensive utilization owing to its prompt onset of action, swift clearance, and rapid patient recovery. Nevertheless, despite the numerous positive attributes displayed by propofol in clinical scenarios, certain constraining factors persist. These encompass potential discomfort at the injection site, the likelihood of encountering issues related to a reduction in blood pressure, and the possibility of adverse effects, such as respiratory depression leading to eventual apnea [32]. Ciprofol has higher lipid solubility than propofol and the concentration of free molecules in the emulsion is significantly lower than propofol, thus potentially causing less injection pain. Studies have shown that HSK3486 acts on the α1β2γ2 receptor subtype and inhibits a broad range of cytochrome P450 isoenzymes in mammals. Its pharmacokinetics and distribution characteristics are rapid metabolism and low accumulation after continuous infusion. Taken together, these findings demonstrate the drug’s strong potential as a clinical alternative to propofol [33]. Presently, there is limited research on ciprofol, although it is recognized as an analogue of propofol. Based on the available studies, it is inferred that ciprofol minimally impacts cortical function. However, beyond its influence on cortical function, ciprofol also affects the hypothalamic-pituitary-adrenal axis [33, 34].

Although ciprofol exhibits superior properties in some aspects, it also has some shortcomings. Ciprofol is metabolized in the body to produce propofol, formaldehyde, and phosphoric acid. Although propofol is its major active metabolite, the formation of formaldehyde may cause local or systemic toxic reactions, which, although usually mild, may cause problems in some sensitive patients. Although ciprofol has better water solubility and reduces the pain during injection, it may still cause adverse reactions at the injection site, such as pain. As a newer drug, ciprofol has relatively little experience and research in clinical use. Although preliminary studies show good results, the safety and effectiveness of long-term use require further research and verification. Ciprofol also costs more and may not be widely available in some areas. This may limit its use in certain medical settings.

Although some combinations of these results achieve statistical significance, they are often reported in only a few studies, making it difficult to draw clinically meaningful conclusions. Our study also has the following limitations: ① Some results are highly heterogeneous. ② Ciprofol is a drug that has just been launched in recent years. There are currently few relevant studies, and most of them are concentrated in China. ③ The data of some studies are quite different, which may be related to the subtle differences in the way outcomes are defined between different studies. Currently, there remains a paucity of clinical studies on ciprofol. Moving forward, larger-scale, multi-center, and comprehensive investigations are imperative to elucidate the merits and drawbacks of ciprofol concerning its anesthetic efficacy, potential side effects, and optimal dosage.

## Conclusion

As a new anesthetic drug, ciprofol has a lower risk of injection-site pain and respiratory depression than propofol both inside and outside the operating room. Ciprofol reduces risk of intraoperative drug-related hypotension. Ciprofol reduces the risk of intraoperative drug-related hypotension and may also reduce the risk of intraoperative body movements. However, ciprofol may have a longer induction time and awakening time than propofol. In addition, the time to disappearance of eyelash reflex or loss of consciousness may also be longer with ciprofol than with propofol. There was no significant difference between the two groups regarding the risk of intraoperative drug-related bradycardia.

### Electronic supplementary material

Below is the link to the electronic supplementary material.

Supplementary Material 1

Supplementary Material 2

Supplementary Material 3

Supplementary Material 4

Supplementary Material 5

Supplementary Material 6

Supplementary Material 7

Supplementary Material 8

Supplementary Material 9

Supplementary Material 10

Supplementary Material 11

Supplementary Material 12

Supplementary Material 13

Supplementary Material 14

Supplementary Material 15

Supplementary Material 16

Supplementary Material 17

Supplementary Material 18

Supplementary Material 19

Supplementary Material 20

## Data Availability

All data generated or analysed during this study are included in this published article [and its supplementary information files].
