# Supplementary figures and images for: Comparison of the efficacy and safety of ciprofol and propofol in sedating patients in the operating room and outside the operating room: a meta-analysis and systematic review
Source: BMC Anesthesiol. 2024 Jul 2;24:218. doi: 10.1186/s12871-024-02609-3 (PMC11218179; doi:10.1186/s12871-024-02609-3)

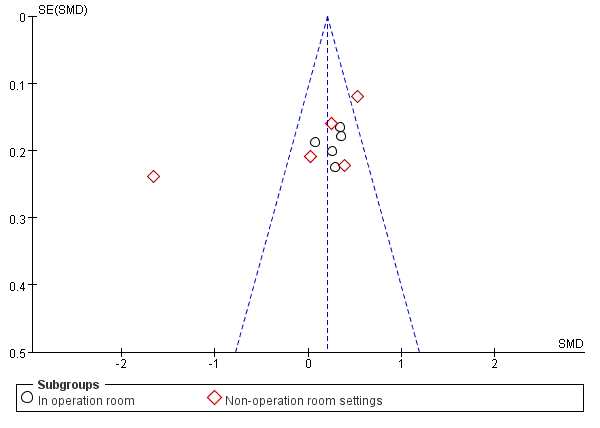

Supplement: Supplementary file 1 — Supplementary Material 1 [file 12871_2024_2609_MOESM1_ESM.png]

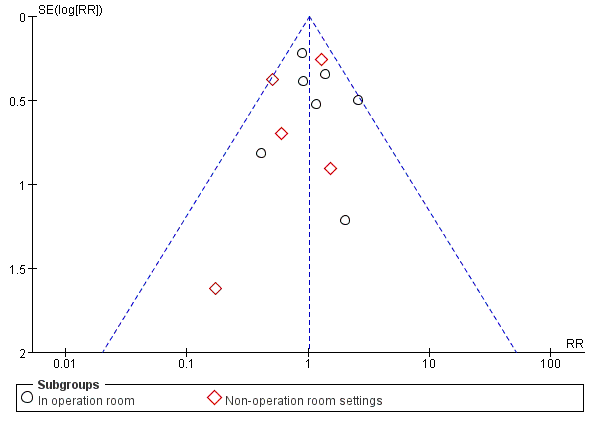

Supplement: Supplementary file 2 — Supplementary Material 2 [file 12871_2024_2609_MOESM2_ESM.png]

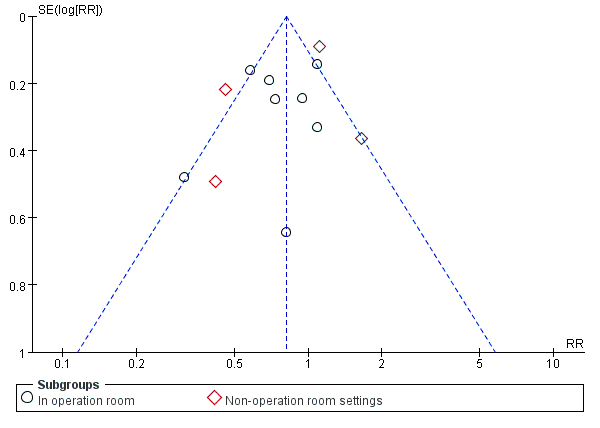

Supplement: Supplementary file 3 — Supplementary Material 3 [file 12871_2024_2609_MOESM3_ESM.png]

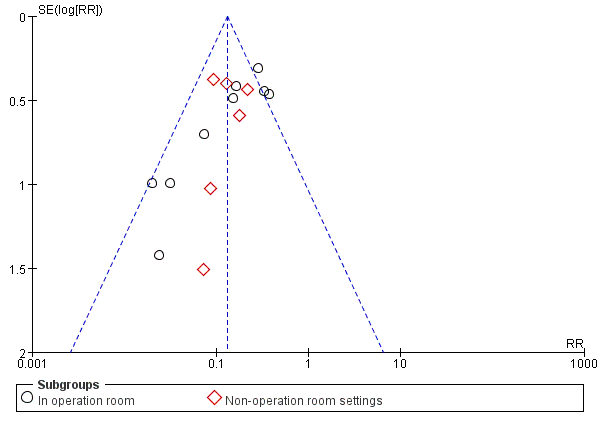

Supplement: Supplementary file 4 — Supplementary Material 4 [file 12871_2024_2609_MOESM4_ESM.png]

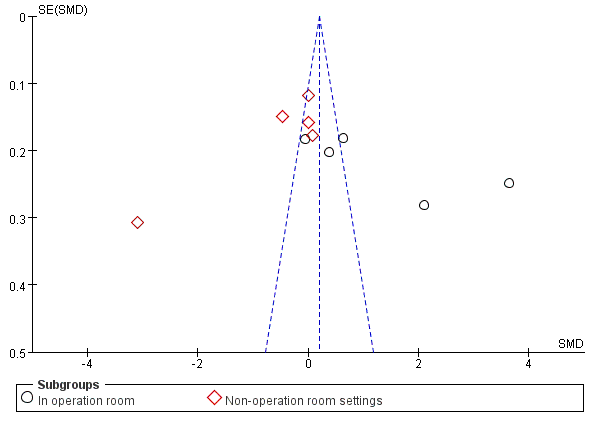

Supplement: Supplementary file 5 — Supplementary Material 5 [file 12871_2024_2609_MOESM5_ESM.png]

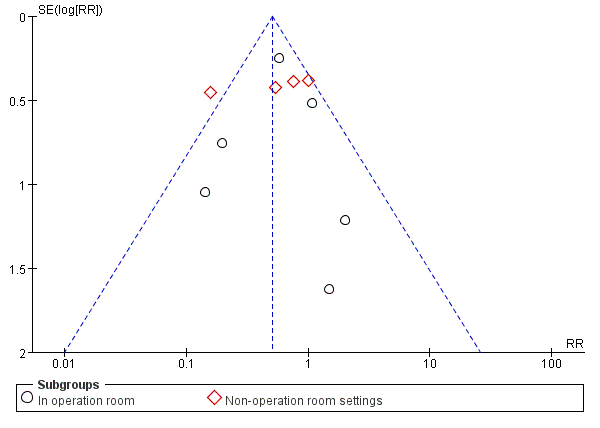

Supplement: Supplementary file 6 — Supplementary Material 6 [file 12871_2024_2609_MOESM6_ESM.png]

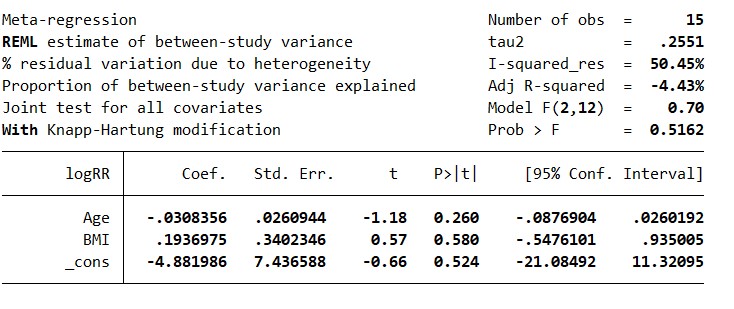

Supplement: Supplementary file 7 — Supplementary Material 7 [file 12871_2024_2609_MOESM7_ESM.jpg]

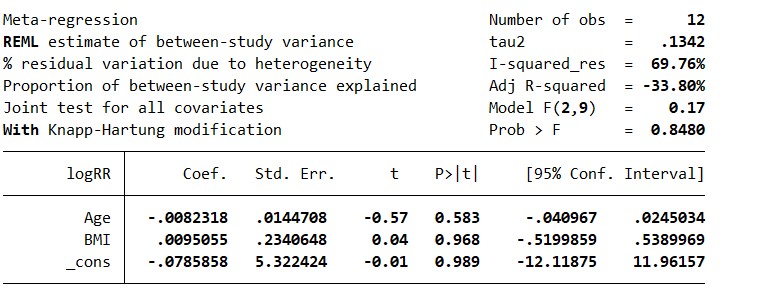

Supplement: Supplementary file 8 — Supplementary Material 8 [file 12871_2024_2609_MOESM8_ESM.jpg]

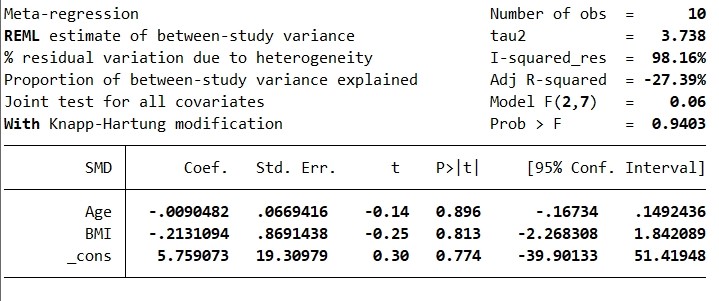

Supplement: Supplementary file 9 — Supplementary Material 9 [file 12871_2024_2609_MOESM9_ESM.jpg]

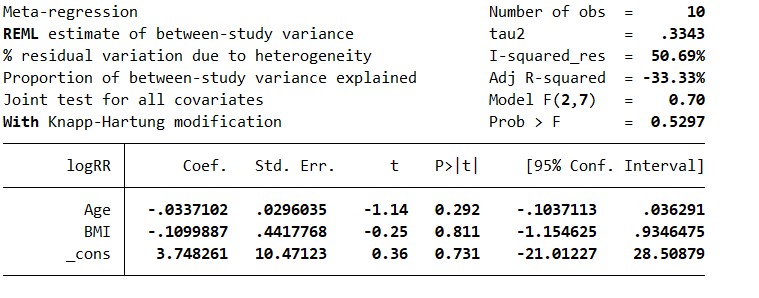

Supplement: Supplementary file 10 — Supplementary Material 10 [file 12871_2024_2609_MOESM10_ESM.jpg]

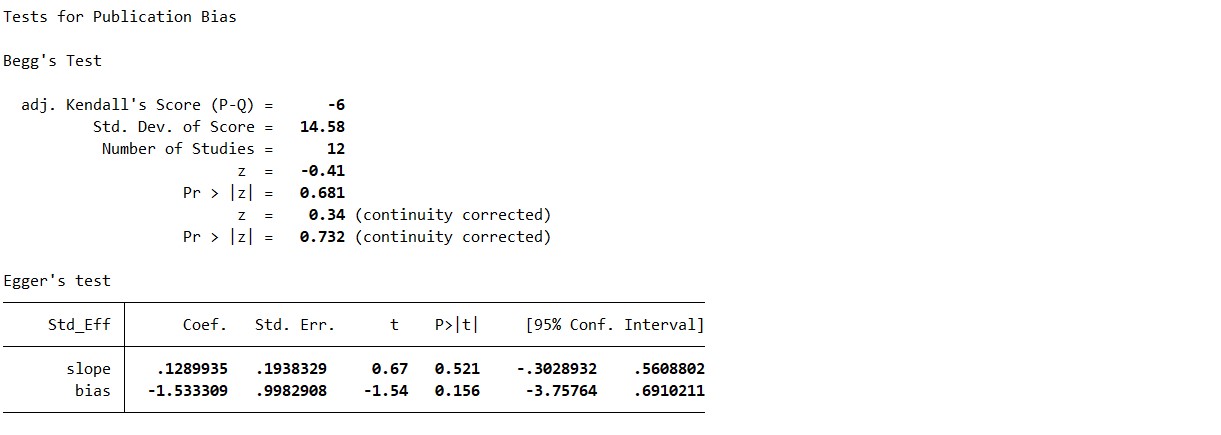

Supplement: Supplementary file 12 — Supplementary Material 12 [file 12871_2024_2609_MOESM12_ESM.jpg]

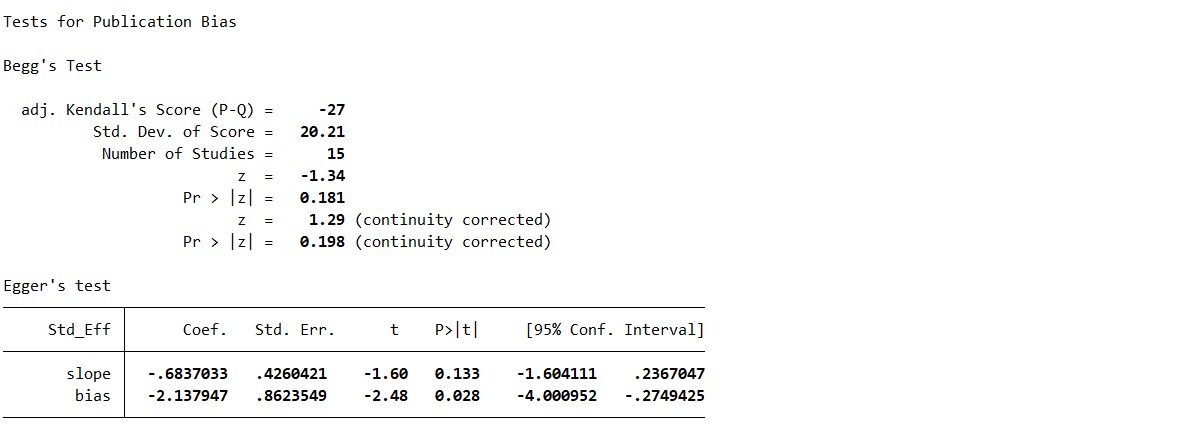

Supplement: Supplementary file 13 — Supplementary Material 13 [file 12871_2024_2609_MOESM13_ESM.jpg]

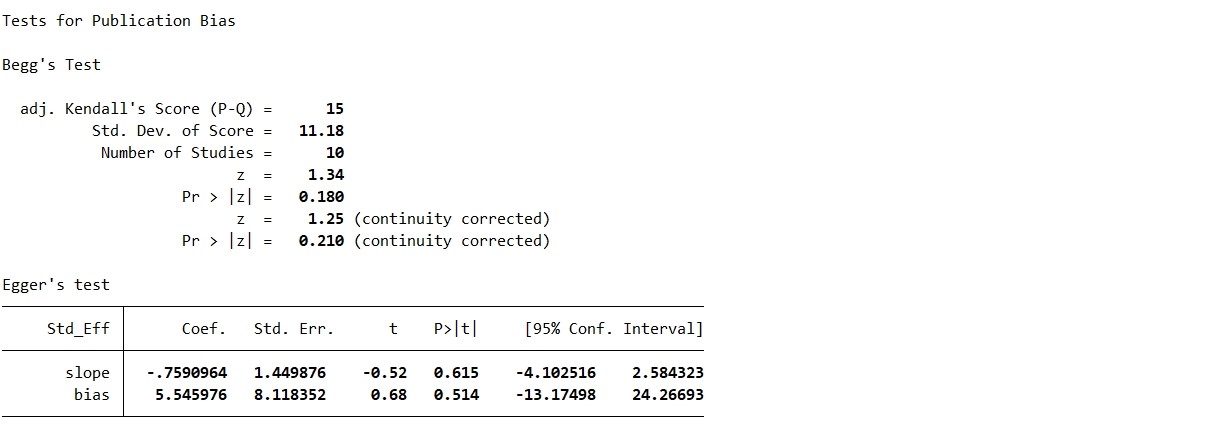

Supplement: Supplementary file 14 — Supplementary Material 14 [file 12871_2024_2609_MOESM14_ESM.jpg]

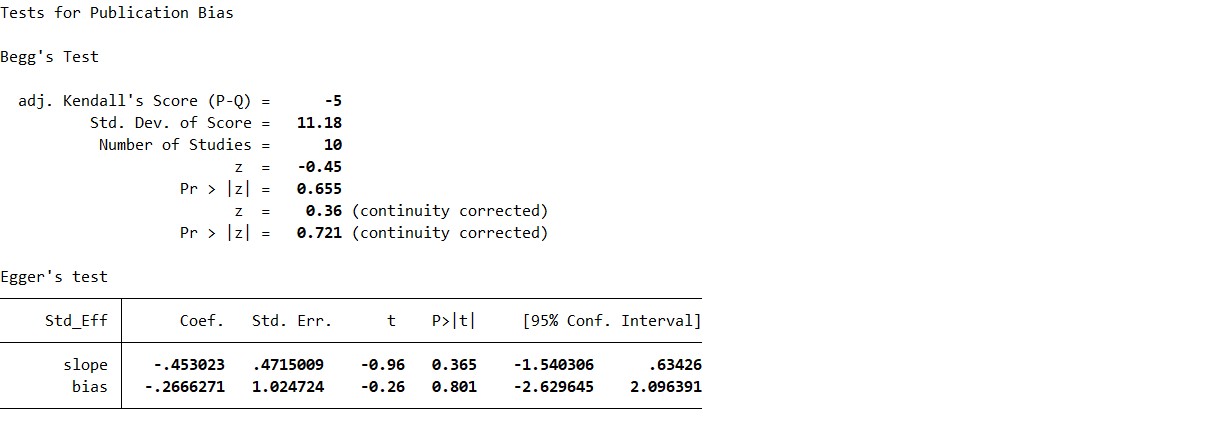

Supplement: Supplementary file 15 — Supplementary Material 15 [file 12871_2024_2609_MOESM15_ESM.jpg]

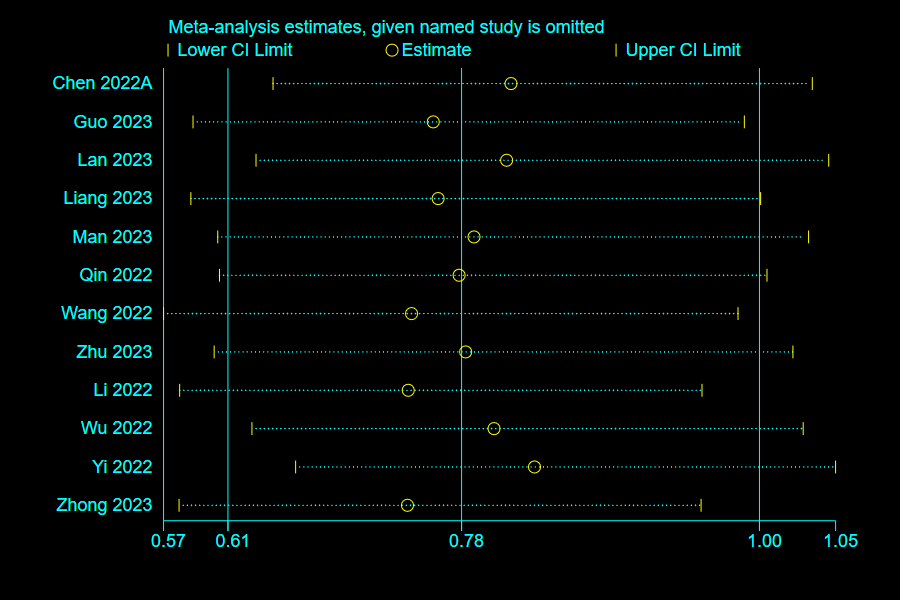

Supplement: Supplementary file 17 — Supplementary Material 17 [file 12871_2024_2609_MOESM17_ESM.tif]

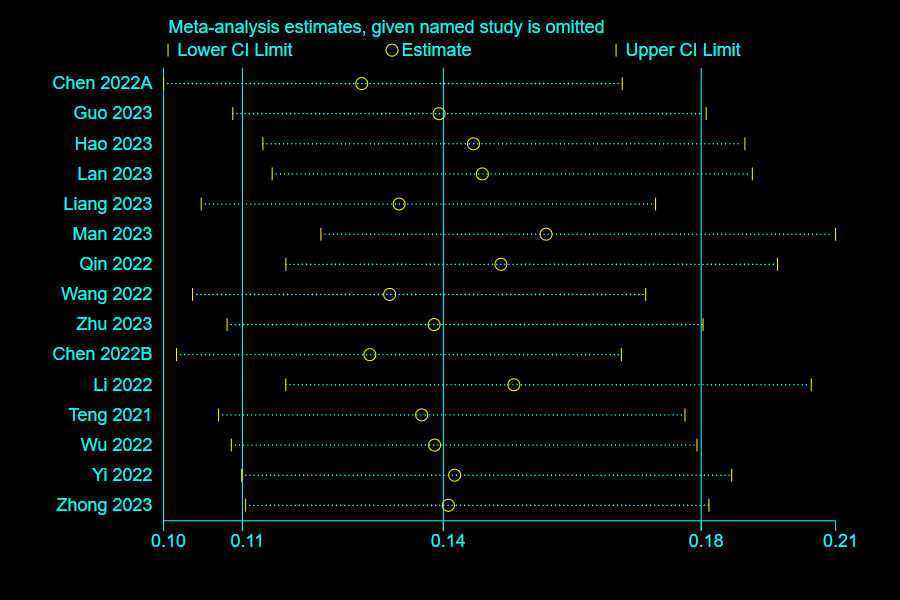

Supplement: Supplementary file 18 — Supplementary Material 18 [file 12871_2024_2609_MOESM18_ESM.tif]

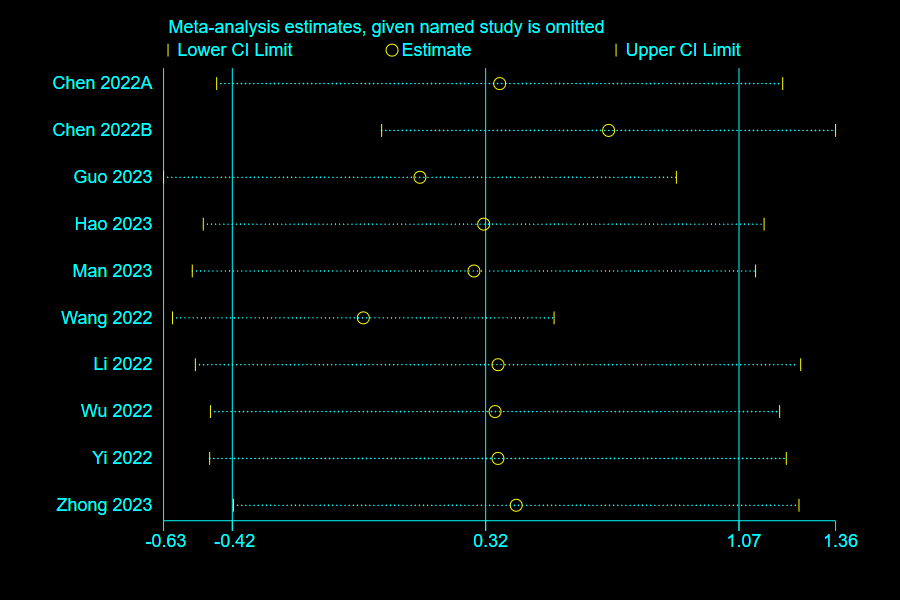

Supplement: Supplementary file 19 — Supplementary Material 19 [file 12871_2024_2609_MOESM19_ESM.tif]

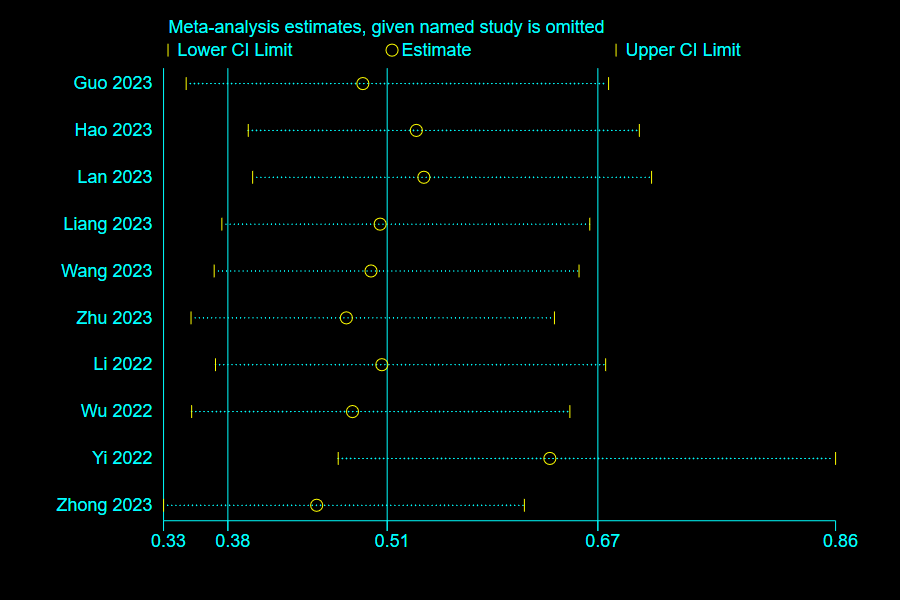

Supplement: Supplementary file 20 — Supplementary Material 20 [file 12871_2024_2609_MOESM20_ESM.tif]
