# Supplementary material for: Comparison of the efficacy and safety of ciprofol and propofol in sedating patients in the operating room and outside the operating room: a meta-analysis and systematic review
Source: BMC Anesthesiol. 2024 Jul 2;24:218. doi: 10.1186/s12871-024-02609-3 (PMC11218179; doi:10.1186/s12871-024-02609-3)
Supplement: Supplementary file 11 — Supplementary Material 11 [file 12871_2024_2609_MOESM11_ESM.docx]

Records identified from:

Embase (n = 68 )

PubMed (n = 127 )

Cochrane Library (n = 104 )

Web of Science (n = 156 )

Records removed before screening:

Duplicate records removed (n = 202 )

**Identification**

Records excluded based on title and abstract (n = 219 )

Records screened (n = 253 )

**Screening**

Reports excluded:

Lack of related data (n = 13 )

Inconsistent research content (n = 6 )

Protocol and abstracts (n = 3 )

Reports assessed for eligibility

(n = 34 )

Studies included in review

**(n = 15 )**

Additional studies were searched (n = 3)

**Included**

*Consider, if feasible to do so, reporting the number of records identified from each database or register searched (rather than the total number across all databases/registers).

**If automation tools were used, indicate how many records were excluded by a human and how many were excluded by automation tools.

*From:*  Page MJ, McKenzie JE, Bossuyt PM, Boutron I, Hoffmann TC, Mulrow CD, et al. The PRISMA 2020 statement: an updated guideline for reporting systematic reviews. BMJ 2021;372:n71. doi: 10.1136/bmj.n71

For more information, visit: <http://www.prisma-statement.org/>
