# Supplementary material for: Comparison of the efficacy and safety of ciprofol and propofol in sedating patients in the operating room and outside the operating room: a meta-analysis and systematic review
Source: BMC Anesthesiol. 2024 Jul 2;24:218. doi: 10.1186/s12871-024-02609-3 (PMC11218179; doi:10.1186/s12871-024-02609-3)
Supplement: Supplementary file 16 — Supplementary Material 16 [file 12871_2024_2609_MOESM16_ESM.docx]

#1 (((((((((((((((((((((("Propofol"[Mesh]) OR (Propofol[Title/Abstract])) OR (2,6-Diisopropylphenol[Title/Abstract])) OR (2,6 Diisopropylphenol[Title/Abstract])) OR (2,6-Bis(1-methylethyl)phenol[Title/Abstract])) OR (Disoprofol[Title/Abstract])) OR (Diprivan[Title/Abstract])) OR (Disoprivan[Title/Abstract])) OR (Fresofol[Title/Abstract])) OR (ICI-35,868[Title/Abstract])) OR (ICI 35,868[Title/Abstract])) OR (ICI35,868[Title/Abstract])) OR (ICI-35868[Title/Abstract])) OR (ICI 35868[Title/Abstract])) OR (ICI35868[Title/Abstract])) OR (Ivofol[Title/Abstract])) OR (Propofol Fresenius[Title/Abstract])) OR (Propofol MCT[Title/Abstract])) OR (Propofol Rovi[Title/Abstract])) OR (Propofol-Lipuro[Title/Abstract])) OR (Recofol[Title/Abstract])) OR (Aquafol[Title/Abstract])) OR (Propofol Abbott[Title/Abstract])

#2 (Ciprofol[Title/Abstract]) OR (hsk3486[Title/Abstract])

#3 #1 AND #2
